# Hysteresis effects of different levels of storm flooding on susceptible enteric infectious diseases in a central city of China

**DOI:** 10.1186/s12889-023-16754-w

**Published:** 2023-09-27

**Authors:** Piao-yi Luo, Meng-xiang Chen, Wen-tao Kuang, Han Ni, Jin Zhao, Hao-yun Dai, Xiang Ren, Shang-hui Yi, Xiu-qin Hong, Wen-ting Zha, Yuan Lv

**Affiliations:** 1 Key Laboratory of Molecular Epidemiology of Hunan Province, Medical School of Hunan Normal University, Changsha, 410000 Hunan China; 2 Changsha Center for Disease Control and Prevention, Changsha, 410000 Hunan China; 3 Hunan Provincial People’s Hospital Affiliated to Hunan Normal University, Changsha, 410000 Hunan China

**Keywords:** Storm flooding, Enteric infectious diseases, Lagged effects, Distributed lag nonlinear model

## Abstract

**Background:**

Recently, attention has focused on the impact of global climate change on infectious diseases. Storm flooding is an extreme weather phenomenon that not only impacts the health of the environment but also worsens the spread of pathogens. This poses a significant challenge to public health security. However, there is still a lack of research on how different levels of storm flooding affect susceptible enteric infectious diseases over time.

**Methods:**

Data on enteric infectious diseases, storm flooding events, and meteorology were collected for Changsha, Hunan Province, between 2016 and 2020. The Wilcoxon Rank Sum Test was used to identify the enteric infectious diseases that are susceptible to storm flooding. Then, the lagged effects of different levels of storm flooding on susceptible enteric infectious diseases were analyzed using a distributed lag nonlinear model.

**Results:**

There were eleven storm flooding events in Changsha from 2016 to 2020, concentrated in June and July. 37,882 cases of enteric infectious diseases were reported. During non-flooding days, the daily incidence rates of typhoid/paratyphoid and bacillary dysentery were 0.3/100,000 and 0.1/100,000, respectively. During flooding days, the corresponding rates increased to 2.0/100,000 and 0.8/100,000, respectively. The incidence rates of both diseases showed statistically significant differences between non-flooding and flooding days. Correlation analysis shows that the best lags for typhoid/paratyphoid and bacillary dysentery relative to storm flooding events may be 1 and 3 days. The results of the distributed lag nonlinear model showed that typhoid/paratyphoid had the highest cumulative RR values of 2.86 (95% CI: 1.71–4.76) and 8.16 (95% CI: 2.93–22.67) after 4 days of general flooding and heavy flooding, respectively; and bacillary dysentery had the highest cumulative RR values of 1.82 (95% CI: 1.40–2.35) and 3.31 (95% CI: 1.97–5.55) after 5 days of general flooding and heavy flooding, respectively.

**Conclusions:**

Typhoid/paratyphoid and bacillary dysentery are sensitive enteric infectious diseases related to storm flooding in Changsha. There is a lagging effect of storm flooding on the onset of typhoid/paratyphoid and bacillary dysentery, with the best lagging periods being days 1 and 3, respectively. The cumulative risk of typhoid/paratyphoid and bacillary dysentery was highest at 4/5 days lag, respectively. The higher of storm flooding, the higher the risk of disease, which suggests that the authorities should take appropriate preventive and control measures before and after storm flooding.

**Supplementary Information:**

The online version contains supplementary material available at 10.1186/s12889-023-16754-w.

## Introduction

The uncertainty of climate change is one of the most serious challenges we face today. Warming, rising sea levels, and frequent weather disasters, which have been listed as the "greatest global health threat of the twenty-first century” by the World Health Organization (WHO), are seriously affecting the sustainable development of the ecological environment and humans [1]. Storm flooding, as a type of extreme weather event, has a serious disease and economic burden on people's lives. However, the definition of storm flooding has not been standardized across countries and regions. For this study, storm flooding is referred to as the inundation of urban areas due to heavy precipitation. This phenomenon includes, but is not limited to, short duration heavy rainfall events, localized heavy rainfall events and the resulting flooding. Storm flooding poses a significant environmental challenge for urban areas. In addition to contaminating water sources, storm flooding damages infrastructure and could potentially pose health hazards.

About 40–50% of the total numbers of natural disasters worldwide are accounted for by this type of disaster, as reported by the International Disaster Database (EM-DAT) [2]. Previous studies have shown that over the past 30 years, accumulated economic losses of approximately $200 billion have been incurred globally due to storm flooding. Among the affected population, it affected the livelihoods of 2.8 billion people and killed 200,000 people, far more than all other natural disasters combined [3, 4]. So far, China has been identified as one of the countries with the highest incidence of natural injuries and damage caused by storm flooding [5]. Between 1984 and 2012, the average annual land area affected by heavy rainfall and flooding in China was 9.37 million square kilometers, of which Hunan Province was among the highest in the country in terms of disaster impact [6]. Changsha, located in the northeastern part of Hunan Province, is a region where storm flooding events are frequent. From June 22 to July 2, 2017, the average precipitation in Hunan Province reached 286.9 mm, with the regional rainstorm comprehensive intensity index reaching its highest value since 1951. A total of 73 counties and cities have experienced extreme precipitation events, with 27 of them recording maximum precipitation levels that exceed historical extremes for 10 consecutive days [7]. In the same year, Changsha experienced the longest and most intense rainfall since the beginning of time, and the water level in the Changsha section of the XiangJiang River exceeded its historical peak, which resulted in a high incidence of typhoid/paratyphoid, bacterial dysentery, and other enteric infections [8, 9].

Some epidemiological evidence suggests a relationship between the increased risk of typhoid/paratyphoid, bacillary dysentery and storm flooding [10, 11]. A study adjusting for long-term trends, seasonality, and meteorological variables shows that storm flooding is associated with an increase in typhoid cases, with a risk ratio of 1.46 for a one-week lag [12]. A study by Li et al. found that the risk of developing bacillary dysentery was significantly and negatively associated with the number of consecutive days of flooding per month. Furthermore, the risk of bacteriophage dysentery is much higher in short but severe floods than in long-term normal floods [13].

Nevertheless, in addition to the direct human losses caused by storm flooding, it may also promote the growth and reproduction of pathogens. The susceptibility of a population can be influenced by pathogens interacting with the host immune system. Enteric infections refer to diseases caused by viruses, bacteria, and parasites that are transmitted through the digestive tract, including but not limited to cholera, typhoid/paratyphoid, bacillary dysentery, and various infectious diarrheal diseases [14]. Most infections are caused by the unsanitary standards of daily life, such as drinkable water and diet, which makes it easy to spread viruses. In the initial stages of storm flooding, with varying degrees of bacterial contamination of food-grade water sources, enteric infections are often the first illnesses in the early stages of a disaster. It can be seen that storm flooding has a significant impact on the risk of developing enteric infectious diseases. To the best of our knowledge, there have been no studies conducted on the classification of storm flooding into different scales. Therefore, this study aims to be the first to explore the effects of storm flooding at three different scales, including non-flooding, general flooding, and heavy flooding. We constructed distributed lag nonlinear model (DLNM) to quantitatively assess the lagged effects of different levels of storm flooding events on susceptible enteric infectious diseases from multiple perspectives. Combined with the storm flooding warnings issued by the meteorological department, our findings can provide a crucial basis for enhancing the emergency response mechanism for storm flooding. This can help prevent or reduce the health risks associated with storm flooding. In addition, our research findings can serve as a reference for the authorities concerned in establishing a response mechanism between extreme weather events and infectious diseases.

## Material and methods

### Definition of storm flooding

The grading of storm flooding events is based on the regulations of the National Comprehensive Study Group of Major Natural Disasters of our National Science Committee and the Meteorological Bureau, and adopts the common criteria for grading storm flooding in the south of China [15]. In this study, general flooding is defined as a single day with total daily precipitation ≥ 100 mm, or any 3–6 consecutive days with a sum of total daily precipitation > 80 mm, or 10 consecutive days with a sum of total daily precipitation > 250 mm. 2 consecutive days with a sum of total daily precipitation > 150 mm or 8 consecutive days with a sum of total daily precipitation > 350 mm is defined as heavy flooding.

### Research area

The research area is Changsha, the capital of Hunan Province, in the central part of China, between 111°53′-114°15′ E and 27°51′-28°41′ N, covering a total area of 11,819 square kilometers. The results of the 7th National Census in 2020 show that the resident population of Changsha has reached 1, 049, 7000 [16]. Due to the subtropical monsoon climate, Changsha has abundant rainfall. Especially during the summer and autumn seasons, there are often brief periods of heavy rain, occasionally even reaching the levels of heavy or exceptionally heavy rainfall. The special topography of Changsha, located in a river valley basin with high ground on both sides and low ground in the middle, makes it an area where storm flooding are frequent.

### Sources of information

According to the Law on the Prevention and Control of Infectious Diseases in China, the management of legal infectious diseases is classified into three levels: A, B and C, corresponding to compulsory, strict and surveillance management, respectively. Category A includes only one enteric infectious disease, cholera. Category B includes typhoid/paratyphoid, bacillary dysentery, etc. Category C has other infectious diarrheal, including norovirus, rotavirus, etc. Therefore, this study has selected representative enteric infectious diseases from these three categories. Cholera, which has been largely eradicated in the China, is not included in the scope of this study. Typhoid/paratyphoid is reported in the disease database under one category. Daily case data on enteric infectious diseases in Changsha for the period 2016–2020 were obtained from the database of legally reported infectious diseases of the China Disease Prevention and Control System. The cases in the study were identified according to the diagnostic criteria and management principles of the disease promulgated by the Chinese Ministry of Health and were clinically diagnosed by professional clinicians based on the symptoms and laboratory findings of the suspected cases. Changsha CDC personnel conduct daily summarization, proofreading, and quality control of infectious disease data. Underreporting of enteric infectious diseases during the study period was corrected using the underreporting rate based on the Changsha CDC underreporting survey report [17]. Meteorological data were obtained from the China Meteorological Data Network (http://data.cma.cn), meteorological factors included average daily temperature, average daily relative humidity, total daily precipitation, 24-h sunshine hours, average daily atmospheric pressure and average daily wind speed. The Hunan Provincial Bureau of Statistics provided the population data for Changsha for the period 2016–2020. Proximity-based interpolation was used to interpolate missing values in the original data. Outliers, such as erroneous and highly abnormal data points, are dealt with by retrospective verification. The study was reviewed and approved by the Ethics Review Committee of the Changsha Centre for Disease Control and Prevention. Data analysis is based on population levels, with associations established through infectious disease outbreak report cards.

### Statistical analysis

#### Screening for susceptible enteric infectious diseases and meteorological factors

This study collected data from 2016 to 2020 in Changsha City on storm flooding days, meteorological conditions, and cases of enteric infectious diseases using Microsoft Excel 2010 software. Descriptive and time series methods were used for data analysis. Based on the definition of storm flooding events were classified and summarized. We provided detailed descriptions of storm flooding levels, including year and date of occurrence, duration, average daily precipitation and maximum daily precipitation. On this basis, the storm flooding was classified into three level s: non-flooding, general flooding, and heavy flooding. The Wilcoxon rank sum test of IBM SPSS Statistics 26.0 was applied to compare the differences in daily incidence of enteric infectious diseases and meteorological factors under different classes of storm flooding conditions, respectively. It screened for the enteric infectious diseases that are susceptible to storm flooding, as well as meteorological factors. We did not perform subsequent lag period and DLNM analyses for enteric infectious diseases and meteorological factors that were not statistically associated with storm flooding. The test level α = 0.05.

#### Lag period analysis

Considering the incidence of infectious diseases, the virulence of the pathogens and the epidemiological characteristics, the disease outcome variables are affected by all these exposure factors. In order to describe the lag effect of exposure, the maximum lag period for enteric infectious diseases was set at 30 days in this study [18]. The relationship between storm flooding and susceptible enteric infectious diseases was explored using the cross-correlation function (CCF) in R software. Moreover, the best lag was determined by the number of days of lag that corresponded to the maximum correlation coefficient in order to inform the subsequent studies.

#### Distributed lag nonlinear model (DLNM)

Distributed lag nonlinear model (DLNM) is commonly used in environmental epidemiology to study health effects of extreme weather. The DLNM has the benefit of concurrently describing the distribution of exposure effects and lag dimensions by creating a crossover matrix between exposure variables and lag days, employing a cross-basis function [19, 20]. Specifically, there is a complex, nonlinear relationship between storm flooding events and health impacts, with usually a lag in health effects [21, 22]. In addition, when investigating the association between storm flooding and disease occurrence, several studies found that meteorological factors could confound the results [23, 24]. To mitigate the impact of meteorological factors on the outcomes, we incorporated them as confounding variables in our analysis. Therefore, this study aims to explore the lag effect of different levels of storm flooding on susceptible enteric infectious diseases using DLNM.

The study period selected was from May to September 2016–2020, which can effectively stabilize seasonal effects. Taking into account the pathogenic characteristics and influencing factors of infectious diseases, the daily number of cases of susceptible enteric infections was collected. The date of diagnosis was matched with the meteorological information and storm flooding events of the same day to eliminate bias caused by differences in detection times. Based on the results of the Wilcoxon rank sum test, we identified the enteric infectious diseases and meteorological factors that were statistically significant in relation to storm flooding events and included them in the model as outcome and confounding variables, respectively. The model included dummy variables for both the time variable (time) and the day of the week effect (dow). With reference to previous studies [25, 26], we controlled for confounders and virtual variables using Natural Cubic Spline (NS) with degrees of freedom (df) set to 3.

The model is constructed as follows:

$$\mathrm{Log}\left[\mathrm E\left({\mathrm Y}_{\mathrm t}\right)\right]=\mathrm\alpha+{\upbeta\mathrm{Variable}}_{\mathrm t,1}+\mathrm{NS}\left(\mathrm{weather},\mathrm{df}\right)+\mathrm{NS}\left({\mathrm{dow}}_{\mathrm t},\mathrm{df}\right)+\mathrm{NS}\left({\mathrm{time}}_{\mathrm t},\mathrm{df}\right)$$

where Y_t_ denotes the number of cases of the susceptible enteric infectious disease on day t; α is the intercept, β is the parameter vector of the variable, variable is denoted as a multicategorical variable, where 0 is non-flooding, 1 is general flooding, and 2 is heavy flooding. Variable_t,l_ is the cross-basis matrix used in the model to estimate the nonlinear and lag relationship between storm flooding events and the number of cases of enteric infectious diseases. NS (weather, df) denotes the meteorological confounders controlled by the natural spline function, including the following four elements: daily average temperature, daily average relative humidity, total daily precipitation, and daily average barometric pressure. NS (dow_t_, df) denotes the natural spline function of the week variable to control for the day-of-week effect, and NS (time_t_, df) denotes the natural spline function of the time variable to control for long-term trends. The model with the smallest Akaike Information Criterion (AIC) value is selected by continuously adjusting the model degrees of freedom and conducting sensitivity analyses to determine the best fit. The maximum lag days were determined based on the incubation period of enteric infectious diseases [27, 28], taking into account the growth and reproduction cycles of pathogenic microorganisms. In detail, the lag range is set at 0–8 days for typhoid/paratyphoid and 0–28 days for bacillary dysentery. Meanwhile, we investigated the delayed health consequences of storm flooding events and analyzed the effects under different time delays. 2D contour plot show the single-day effects of different levels of storm flooding-lag days. Furthermore, the cumulative impact of lags over multiple days is taken into account. Cumulative relative risk refers to the total number of risks that occur over a given period of time. Analyzing the effects of storm flooding events on enteric infectious diseases that are susceptible can be more thoroughly assessed by examining both single-day and cumulative impacts. Model was constructed using R3.4.3 software and the DLNM package (version 2.1.3), relative risk (RR) effect sizes were calculated, and the resulting data were visualized. The RR value was used as the effect statistic and when the 95% CI of the RR value was completely above 1, the effect was indicated to be statistically significant. In addition, in practice, the prevalence of disease in an area is influenced by many factors. There is a limitation to the distributed lag nonlinear model. It has difficulty incorporating social factors such as health practices and immunization prophylaxis into the analysis, which affects quantitative estimates of the risk of morbidity.

## Results

### Descriptive analysis

#### Characteristics of the distribution of storm flooding and meteorological factors

**Fig. 1:**
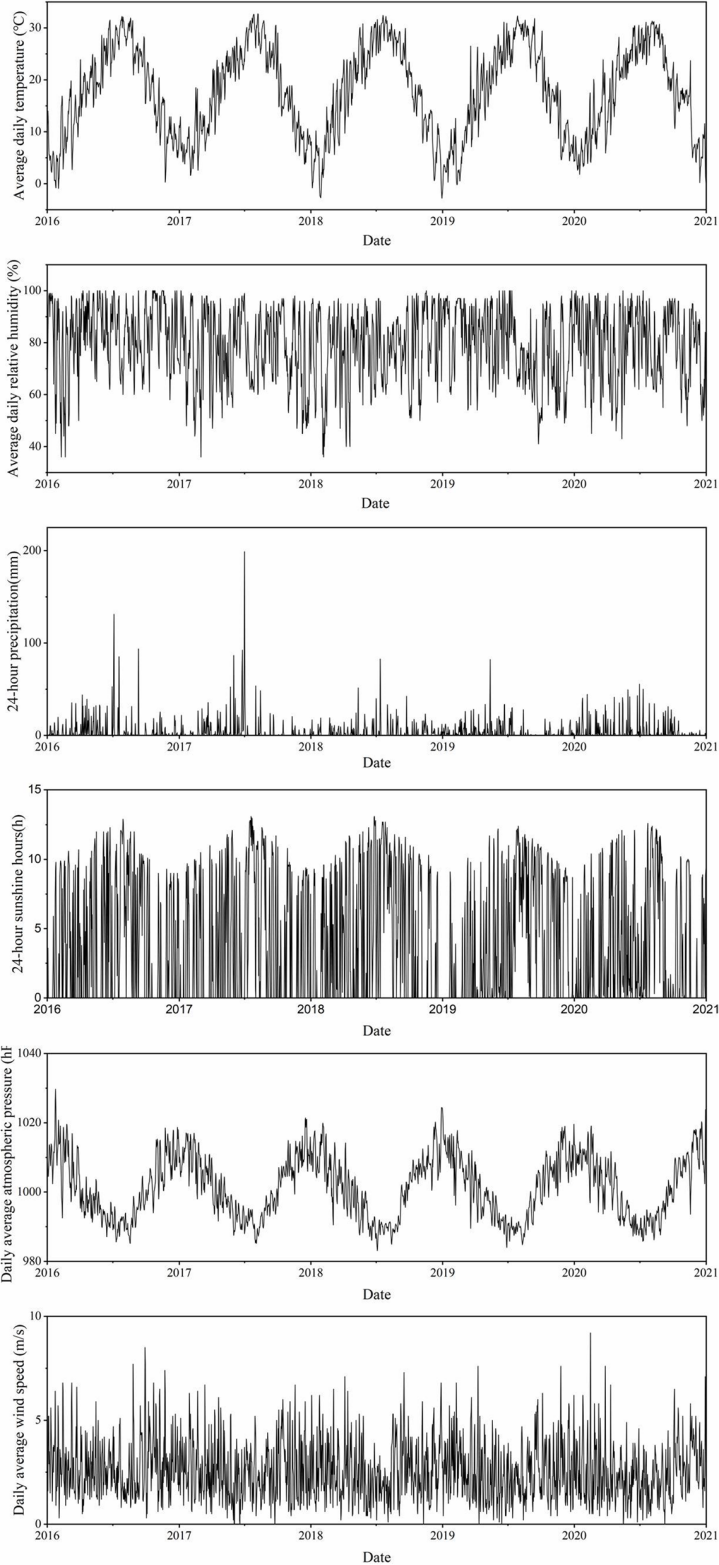
Time series of meteorological factors in Changsha, 2016–2020

Based on the storm flooding criteria, eleven storm flooding events with a total duration of 47 days were recorded in Changsha during the 2016–2020 study periods. The months of occurrence are May to September. General flooding occurred 8 times, 3 times each in 2016 and 2017, and 1 time each in 2018 and 2019. Heavy flooding occurred 3 times, in 2016 (1) and 2017 (2). No storm flooding events occurred in 2020. The frequency of storm flooding decreased each year during the study period (Table 1). The average daily temperature in Changsha for the same period ranged from -2.8 °C to 32.7 °C, with an average daily relative humidity of (79.1 ± 14.1) %. The maximum daily precipitation total is 198.9 mm; 24-h sunshine hours (4.1 ± 4.5) h, daily average atmospheric pressure (1001.4 ± 8.9) hPa, daily average wind speed (2.6 ± 1.4) m/s (Fig. 1).

**Table 1:** Basic information on storm flooding events in Changsha, 2016–2020

		Year	Date (start–end)	Duration (d)	Average daily precipitation (mm)	Maximum daily precipitation (mm)
General flooding	1	2016	7.3–7.8	6	34.8	131.1
	2	2016	7.18–7.20	3	28.4	85.2
	3	2016	9.10–9.12	3	32.2	93.7
	4	2017	6.1—6.3	3	28.9	86.6
	5	2017	6.24–6.27	4	43.5	92.4
	6	2017	6.30–7.9	10	33.5	198.9
	7	2018	7.12–7.14	3	41.4	82.7
	8	2019	5.13–5.15	3	27.5	82.2
Heavy flooding	1	2016	7.4–7.5	2	76.2	131.1
	2	2017	6.25–6.26	2	80.3	92.4
	3	2017	7.1–7.8	8	29.1	198.9

#### Occurrence of enteric infectious diseases

**Fig. 2:**
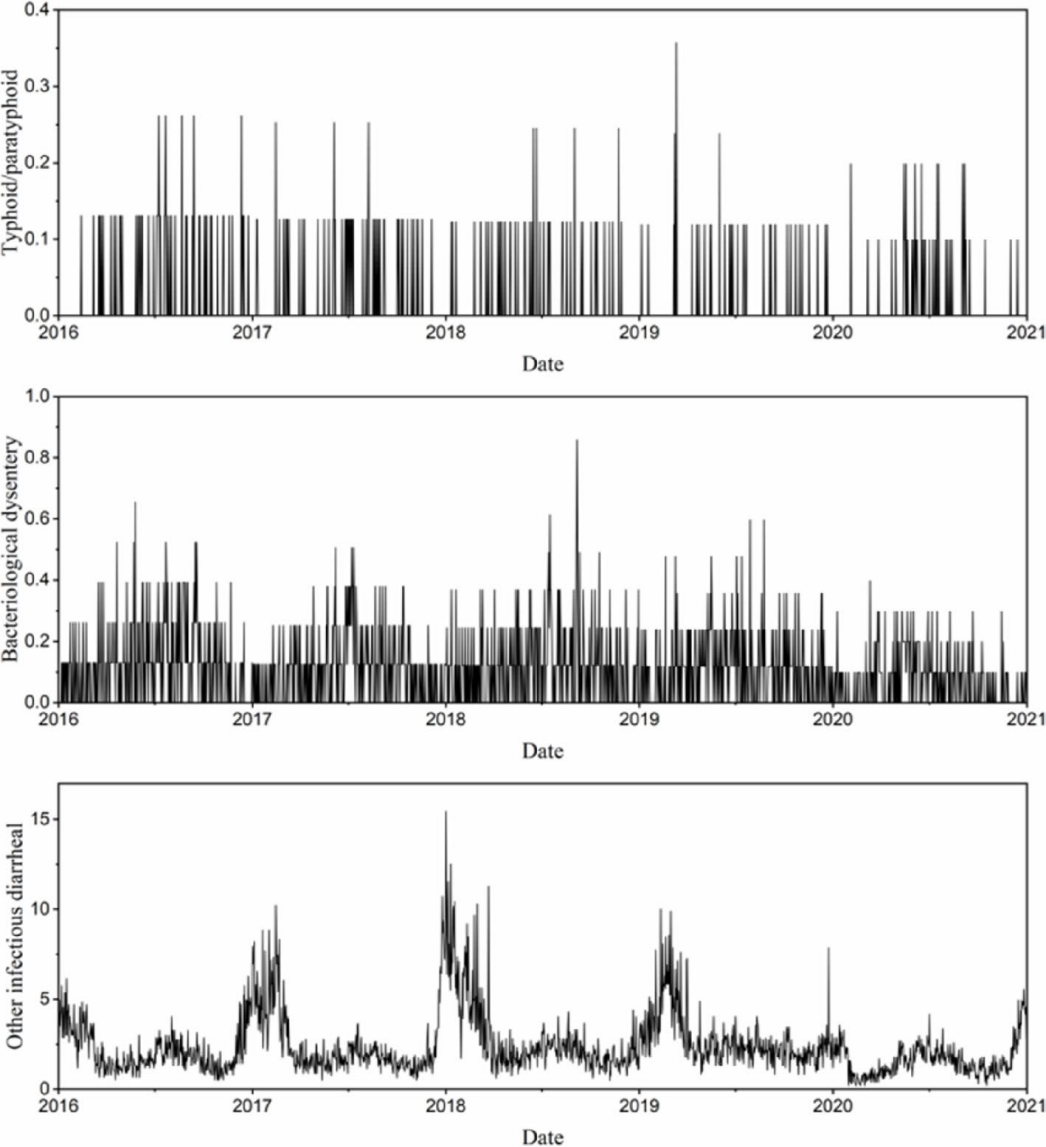
Time series of daily incidence of enteric infectious diseases in Changsha, 2016–2020

From 2016 to 2020, a total of 255 cases of typhoid/paratyphoid were reported in Changsha, with an average annual reported incidence rate of 0.62/100,000, 1,589 cases of bacteriological dysentery were reported, with an average annual reported incidence rate of 3.8/100,000, and 36,038 cases of other infectious diarrheal were reported, with an average annual reported incidence rate of 86.6/100,000 (Table 2). The overall trend in typhoid/paratyphoid incidence is decreasing year by year, the incidence of bacteriological dysentery first decreased and then increased, followed by a decreasing trend year by year, the incidence of other infectious diarrheal first increased and then decreased. The incidence of enteric infectious diseases in the period of high storm flooding (May to September) showed that the incidence of typhoid/paratyphoid and bacillary dysentery was higher than that of other infectious diarrheal. However, the peak of enteric infectious diseases in the non-flooding period occurred in the winter of 2019 (Fig. 2).

**Table 2:** Incidence rates of enteric infectious diseases in Changsha, 2016–2020

Year	Permanent residence population (× 10^4^ people)	Typhoid / paratyphoid		Bacterial dysentery		Other infectious abdominal diseases	
		Number of cases	Incidence rate ^a^	Number of cases	Incidence rate ^a^	Number of cases	Incidence rate ^a^
2016	764.52	63	0.824	335	4.381	5954	77.879
2017	791.81	56	0.707	309	3.902	7315	92.383
2018	815.47	49	0.601	354	4.341	8418	103.229
2019	839.45	39	0.465	341	4.062	8500	101.257
2020	1006.08	48	0.477	250	2.485	5851	58.156

^a^ Incidence rate (1/100,000)

### Screening for susceptible enteric infectious diseases and meteorological factors

In terms of daily incidence, typhoid/paratyphoid had 0.3/100,000 and 0.1/100,000, while bacillary dysentery had 2/100,000 and 0.8/100,000 on flooding and non-flooding days, respectively. The results of the Wilcoxon rank sum test shows that the differences between the daily incidence rates of typhoid/paratyphoid and bacillary dysentery are statistically significant on non-flooding days compared with general flooding days and heavy flooding days, with *P* < 0.001. The differences between the daily incidence rates of other enteric infectious diseases on non-flooding days and general flooding days and heavily flooding days are not statistically significant, with *P* > 0.05. There were differences in daily mean temperature and daily mean air pressure between non-flooding days and general flooding days and heavily flooding days, and differences in daily mean relative humidity and total daily precipitation between non-flooding days and general flooding days, and the differences were statistically significant, with *P* < 0.001.

The findings suggest that the occurrence of storm flooding events may influence the spread of typhoid/paratyphoid and bacillary dysentery, which can be considered as storm-flooding-sensitive diseases in Changsha. The results also suggest that there is significant variability between storm flooding and four of the meteorological factors (average daily temperature, average daily atmospheric pressure, average daily relative humidity and total daily precipitation) (Table 3).

**Table 3:** Two-by-two comparison of enteric infectious diseases and meteorological factors in Changsha, 2016–2020

Variable	Two-by-two comparison		Z	*P*
Diseases				
Typhoid/paratyphoid	Non-flooding	General flooding	6.845	< 0.001
	Non-flooding	Heavy flooding	6.133	< 0.001
	General flooding	Heavy flooding	0.537	0.591
Bacterial dysentery	Non-flooding	General flooding	6.698	< 0.001
	Non-flooding	Heavy flooding	4.859	< 0.001
	General flooding	Heavy flooding	0.440	0.660
Other infectious diarrhea	Non-flooding	General flooding	0.900	0.368
	Non-flooding	Heavy flooding	1.102	0.271
	General flooding	Heavy flooding	0.418	0.676
Meteorological factor				
Average daily temperature (℃)	Non-flooding	General flooding	3.987	< 0.001
	Non-flooding	Heavy flooding	3.547	< 0.001
	General flooding	Heavy flooding	1.043	0.297
Average daily relative humidity (%)	Non-flooding	General flooding	3.479	< 0.001
	Non-flooding	Heavy flooding	1.793	0.073
	General flooding	Heavy flooding	0.435	0.663
Total daily precipitation (mm)	Non-flooding	General flooding	3.692	< 0.001
	Non-flooding	Heavy flooding	1.454	0.146
	General flooding	Heavy flooding	0.461	0.645
24-h sunshine hours(h)	Non-flooding	General flooding	1.172	0.241
	Non-flooding	Heavy flooding	0.515	0.607
	General flooding	Heavy flooding	1.140	0.254
Daily average atmospheric pressure (hPa)	Non-flooding	General flooding	5.255	< 0.001
	Non-flooding	Heavy flooding	4.103	< 0.001
	General flooding	Heavy flooding	0.052	0.958
Daily average wind speed (m/s)	Non-flooding	General flooding	0.964	0.335
	Non-flooding	Heavy flooding	0.464	0.642
	General flooding	Heavy flooding	1.096	0.273

### Lag period

The lag days corresponding to the maximum number of correlations were initially set as the optimal lag period. The results showed that the correlation between storm flooding events and the incidence of typhoid/paratyphoid and bacillary dysentery was highest on day 1 and day 3, respectively. As a result, the optimal time lags correspond to days 1 and 3, respectively (Figure S1 in the Supplementary Information).

### DLNM analysis

#### Single-day effects of storm flooding on typhoid/paratyphoid and bacillary dysentery with different lag days

**Fig. 3:**
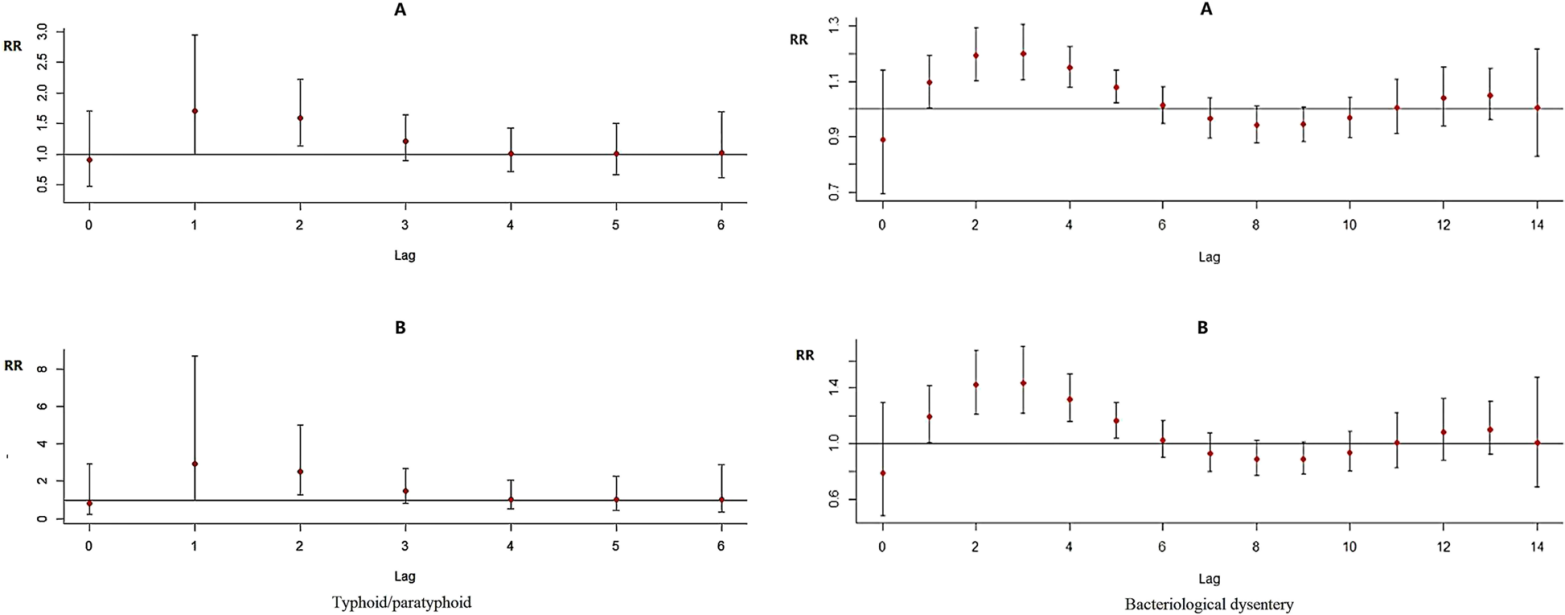
Relationship between storm flooding and Ty/Pty, BD (**A**: general flooding, **B**: heavy flooding)

The results of the study indicate that both general flooding and heavy flooding significantly increase the risk of typhoid/paratyphoid and bacillary dysentery. The relationship between storm flooding and susceptible enteric infectious diseases is roughly S-shaped. Of these, the relationship between storm flooding and typhoid/paratyphoid showed a significant impact on infection risk at lag day 1, with the lag effect persisting until day 2. The optimal lag period is day 1, when the risk of typhoid/paratyphoid peaks. The relationship between storm flooding and bacillary dysentery showed a statistically significant risk of morbidity during lag days 1 to 5. The highest risk of bacillary dysentery was observed on day 3 of the lag period. As the delay increased, the risk of typhoid/paratyphoid and bacillary dysentery due to storm flooding decreased to insignificance (Fig. 3, Figure S2 in the Supplementary Information).

#### Cumulative effects of storm flooding on typhoid/paratyphoid and bacillary dysentery with different lag days

The cumulative risk of typhoid/paratyphoid and bacillary dysentery was highest at 4/5 days lag, respectively. After this, the cumulative morbidity risk gradually decreases as the number of days lagged increases. The cumulative RRs for typhoid/paratyphoid are 2.86 (95% CI: 1.71–4.76) and 8.16 (95% CI: 2.93–22.67) for general flooding and heavy flooding lags 0–4 days, respectively, the cumulative risks for lags 0–7 days are 2.29 (95% CI: 1.09–4.79) and 5.23 (95% CI: 1.19- 22.91), respectively, and the cumulative risks for lags 0–8 days are 1.64 (95% CI: 0.70–3.85) and 2.69 (95% CI: 0.49–14.85), respectively. The effect of storm flooding on the onset of typhoid/paratyphoid lasted for about 1 week.

The cumulative RR values for bacillary dysentery at 0–5 days lag are 1.82 (95% CI: 1.40–2.35) and 3.31 (95% CI: 1.97–5.55) for general flooding and heavy flooding, respectively; 1.60 (95% CI: 1.14–2.24) and 2.57 (95% CI: 1.31–5.03) for 0–14 days lag; The cumulative RR values for lag 0–29 days were 1.67 (95% CI: 0.99–2.82) and 2.79 (95% CI: 0.98–7.96), respectively. This shows that the impact of storm flooding on the onset of bacterial dysentery will dissipate for about 1 month. Under different levels of storm flooding conditions, the impact of the heavy flooding on typhoid/paratyphoid and bacillary dysentery was more significant.

## Discussions

This study investigated the impact of storm flooding on susceptible enteric infections in Changsha and found that typhoid/paratyphoid was a susceptible enteric infection to storm flooding events during the period 2016–2020. Some counties (districts) in China have high incidence rates, even reaching the incidence levels of moderately endemic areas. Among them, the incidence of typhoid/paratyphoid in Hunan is much higher than the national [29]. The risk of typhoid/paratyphoid exists almost all year round. Especially in February each year, the number of typhoid and paratyphoid incidences begins to increase gradually [30]. This is most likely due to the increased rainfall and relative humidity of the air during this period, which increases the likelihood and amount of contamination of water or food with pathogens [31, 32].

After conducting further analysis of the lag effect of storm flooding on enteric infectious diseases, it is indicated that the day 1 lag period was found to be the optimal time for the onset of typhoid/paratyphoid concurrent with storm flooding. This finding may be attributed to the relatively short incubation period of the disease, which typically spans from 5 to 9 days. Following infrastructure damage caused by storm flooding, the subsequent damage to manure storage pipes and sewers may result in the contamination of food or water with domestic sewage and garbage. Most people are infected by the pathogen after ingestion [33]. Similar results have been obtained in a community-based case–control study conducted in Indonesia, where storm flooding damage to households was found to be a risk factor for the development of typhoid/paratyphoid (OR = 4.52) [34]. However, some studies have also shown no increase in the incidence of typhoid/paratyphoid after a disaster [35]. The relationship between storm flooding and typhoid/paratyphoid is still somewhat controversial. This may be related to regional differences in studies, the influence of other factors. Meanwhile, we found that the cumulative RR values were 2.86 (95% CI: 1.71–4.76) and 8.16 (95% CI: 2.93–22.67) 4 days after the occurrence of general flooding and heavy flooding, respectively. This indicates that the risk of typhoid/paratyphoid incidence increases with more severe storm flooding. This implies that as storm flooding becomes more severe, we need to pay more attention to typhoid/paratyphoid prevention and control.

This study identified bacillary dysentery as a sensitive enteric disease to storm flooding in Changsha. Bacillary dysentery exhibits a seasonal pattern with its peak incidence occurring during summer and autumn (May to September). It is primarily transmitted by Shigella and is among the most common causes of diarrheal illness [36]. Shigella multiplies in the enteric tract of patients with acute and chronic dysentery and is excreted in the faeces. This phenomenon may occur because people are often infected by eating or drinking food or water contaminated with Shigella. The change in the growth conditions of Shigella is due to the heavy precipitation during storm flooding, which makes the air more conducive to the growth and reproduction of the pathogen when the temperature and humidity are suitable [13]. This study concluded that its optimal lag period was day 3. It is worth noting that the results of both the cross-correlation function and distributed lag nonlinear model suggest that storm flooding has short-term lag effects on typhoid/paratyphoid and bacillary dysentery. The cumulative effect of bacillary dysentery peaked on day 5, with cumulative RR values of 1.82 (95% CI: 1.40–2.35) and 3.31 (95% CI: 1.97–5.55) for general flooding and severe flooding, respectively. Most studies concluded that the impact of storm flooding on bacteriological dysentery in Hunan Province from 2004–2011 was greatest at a lag of 1 week (RR = 1.12, 95% CI: 1.05–1.20) [37]. In the north-central part of Henan Province, the storm flooding months increased the incidence of bacteriological dysentery (RR = 1.66, 95% CI: 1.52–1.82) [21]. Another study reminds people that the storm flooding time is prolonged and the intensity of rainfall increases, which will lead to higher incidence of enteric infectious diseases [38]. Thus, storm flooding is indeed an important factor in increasing the incidence of bacteriological dysentery. In addition, this study also found that the effect of storm flooding on the incidence of bacteriophage dysentery lasted for about 1 month. The effect of storm flooding on bacteriophage dysentery in Hunan Province in 2007 lasted for nearly 1 month after a storm flooding event, which is similar to the results of this study [39]. This suggests that our preventive and control measures for bacillary dysentery during storm flooding last at least one month to better avoid the negative effects of storm flooding. Our study also shows that the high incidence of enteric infectious diseases during non-flooding mainly occurred in winter. This finding is consistent with the study by Meakins SM et al. [40, 41]. The incidence of these diseases tends to increase during the winter months, likely due to the cold weather and increased indoor activity. It is easier to spread among the crowd.

As climate instability becomes more severe in the future, our study can provide a more effective theoretical support for public health practice policy in Changsha. Firstly, the timeliness and authenticity of infectious disease surveillance reports should be comprehensively strengthened; secondly, the supply of clean drinking water and food should be ensured, medical relief work should be co-ordinated, and immunization of vulnerable populations should be effectively carried out; finally, timely scientific knowledge about prevention and control of infectious diseases should be disseminated to strengthen the awareness of prevention among residents in the affected areas. Our findings can provide data support and scientific basis for the formulation of prevention and control plans for infectious diseases after storm flooding. It is of great significance in reducing the negative health effects brought about by extreme weather.

## Innovations and limitations

The advantage of applied distribution lag nonlinear model over linear model is that it allows more flexibility in exploring the nonlinear relationship between storm flooding and infectious disease. Secondly, in order to investigate the effect of storm flooding on disease more precisely, the lag period was added in this study. By finding the optimal lag period, it provides a basis for subsequent studies. In addition, data from the months with the highest frequency of storm flooding event (May to September) were selected for analysis in this study, thus effectively controlling for seasonality.This makes the results more convincing.

This study also has some shortcomings. First of all, it did not take into account the differences between urban and rural areas. When responding to storm flooding, there are huge differences in living standards and medical facilities between urban and rural areas, resulting in different levels of contamination of food and water sources [42, 43]. Secondly, in subsequent studies, we should try to incorporate multiple factors affecting the incidence of infectious diseases into the model and continuously adjust the variables and their parameters in the model in order to observe changes in the RR that are closer to the actual situation.

## Conclusion

In summary, typhoid/paratyphoid and bacillary dysentery are sensitive enteric infectious diseases related to storm flooding in Changsha. It is therefore important to promote awareness among the population by disseminating information on the prevention and control of related infectious diseases before storm flooding. As there is a lag period for the onset of typhoid/paratyphoid and bacillary dysentery due to storm flooding, the optimal lag periods are day 1 and day 3 respectively. This could inform the health authorities in advance to develop contingency plans to reduce the prevalence of the disease. Meanwhile, our study found that the cumulative risk of typhoid/paratyphoid and bacillary dysentery was highest at 4/5 days lag, respectively. The effect of storm flooding on the onset of typhoid/paratyphoid lasts for about 1 week and the effect on the onset of bacillary dysentery will dissipate in about 1 month. The higher the level of storm flooding, the higher the risk of disease outbreaks. This suggests that the authorities should carry out timely routine immunization and take appropriate preventive and control measures before and after storm flooding.

### Supplementary Information

**Additional file 1: Figure S1.** Correlation analysis of enteric infectious diseases and storm flooding (A: typhoid/paratyphoid, B: bacteriological dysentery). **Figure S2.** Risk of different levels of storm flooding and lag days on the onset of Ty/Pty and BD.

## Data Availability

The datasets used and/or analyzed during the current study available from the corresponding author on reasonable request.

## Abbreviations

CCF: Cross-correlation Function
DLNM: Distributed lag nonlinear model
Ty/Pty: Typhoid/paratyphoid
BD: Bacteriological dysentery
