# Supplementary material for: Hysteresis effects of different levels of storm flooding on susceptible enteric infectious diseases in a central city of China
Source: BMC Public Health. 2023 Sep 27;23:1874. doi: 10.1186/s12889-023-16754-w (PMC10537077; doi:10.1186/s12889-023-16754-w)
Supplement: Supplementary file 1 — Additional file 1: Figure S1. Correlation analysis of enteric infectious diseases and storm flooding (A: typhoid/paratyphoid, B: bacteriological dysentery). Figure S2. Risk of different levels of storm flooding and lag days on the onset of Ty/Pty and BD. [file 12889_2023_16754_MOESM1_ESM.docx]

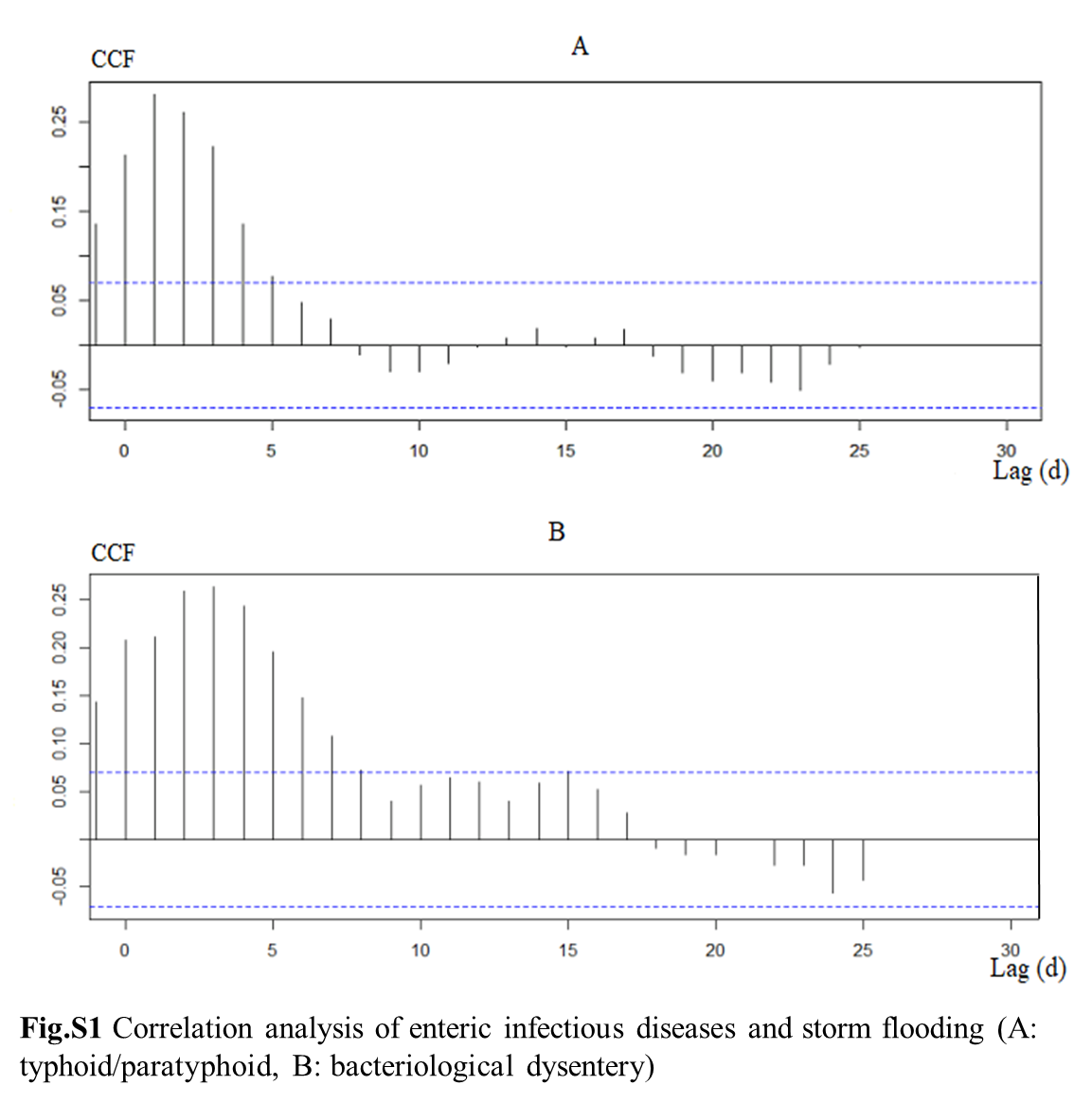


Figure S1 Correlation analysis of enteric infectious diseases and storm flooding (A: typhoid/paratyphoid, B: bacteriological dysentery)


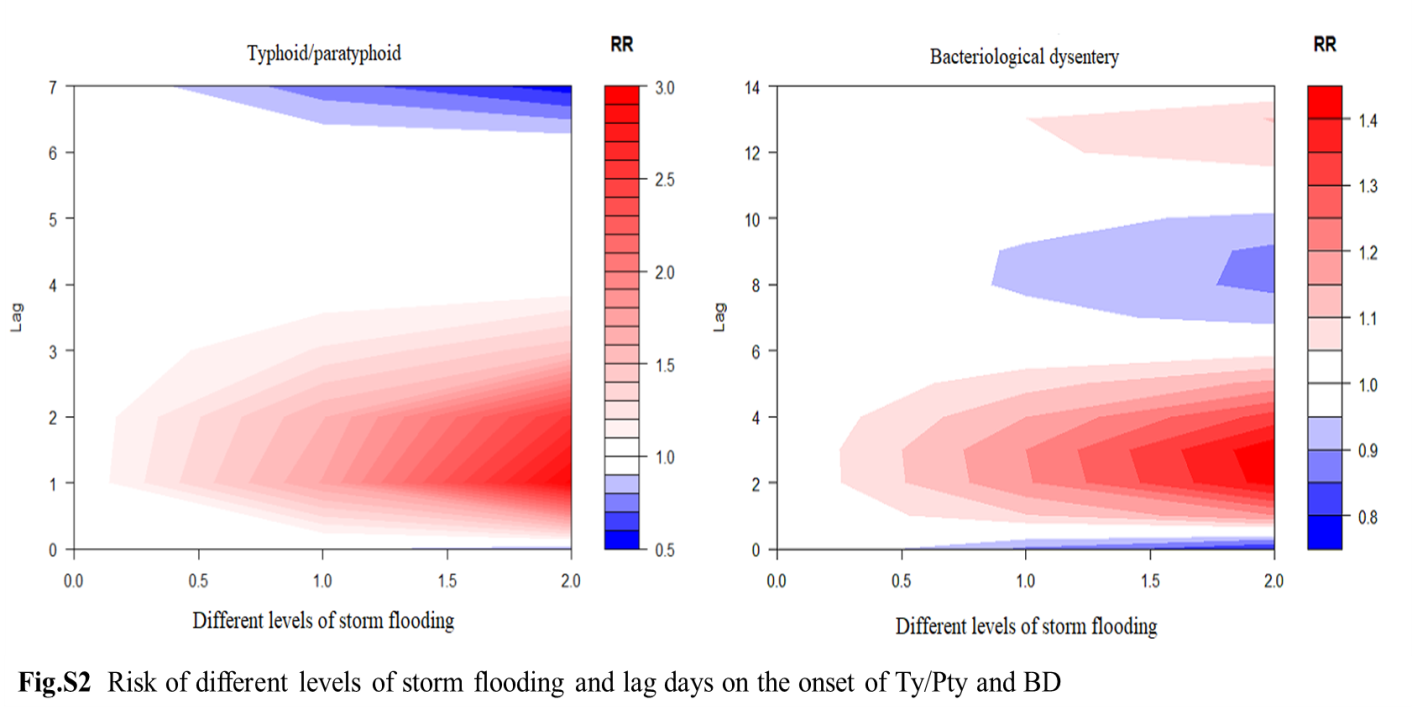


Figure S2 Risk of different levels of storm flooding and lag days on the onset of Ty/Pty and BD
